# Editorial: Meiotic Recombination and DNA Repair: New Approaches to Solve Old Questions in Model and Non-model Plant Species

**DOI:** 10.3389/fpls.2022.841402

**Published:** 2022-02-09

**Authors:** Olivier Da Ines, Kyuha Choi, Mónica Pradillo, Christophe Lambing

**Affiliations:** ^1^Institut Génétique Reproduction et Développement (iGReD), Université Clermont Auvergne, Centre National de la Recherche Scientifique (CNRS), Institut national de la santé et de la recherche médicale (INSERM), Clermont-Ferrand, France; ^2^Department of Life Sciences, Pohang University of Science and Technology, Pohang, South Korea; ^3^Department of Genetics, Faculty of Biology, Physiology and Microbiology, Universidad Complutense de Madrid, Madrid, Spain; ^4^Rothamsted Research, Harpenden, United Kingdom

**Keywords:** meiosis, epigenetics, genome evolution, DNA double strand break, homologous recombination

Accurate segregation of chromosomes at the first meiotic division relies upon the establishment of physical connections between homologous chromosomes, which with a few exceptions, are realized by crossover recombination. Recombination also reshuffles genetic information between homologs, and thus strongly influences genome evolution. At the molecular level, meiotic recombination is initiated by the programmed induction of DNA double strand breaks (DSBs) and their subsequent repair as a crossover (CO) or a non-crossover (NCO). However, COs are constrained and the majority of DSBs are repaired as NCOs in plants. Gutierrez Pinzon et al. provide a comprehensive overview of the most recent findings on the different steps controlling meiotic recombination, with an emphasis on the different anti-CO pathways. Notably, one of these, involving the RecQ4 helicase, has previously been shown to be active in Arabidopsis, rice, pea and tomato (Séguéla-Arnaud et al., 2015; Mieulet et al., 2018). Arrieta et al. extend this anti-CO role to the cereal barley. Through a suppressor screen of a CO-defective mutant, they show that mutating the *RecQ4* gene in Barley can increase meiotic recombination by nearly two-fold. The RecQ4 anti-CO pathway, initially discovered in Arabidopsis, appears thus largely conserved and translatable to cereals. Mechanisms of meiotic recombination are thus largely conserved across plant kingdom. Nevertheless specificities exist, as nicely illustrated by the characterization of the maize checkpoint clamp loader RAD17 by Zhang et al. RAD17 is not essential for meiotic DSB repair in Arabidopsis, while rice *Osrad17* mutants exhibit extensive meiotic chromosome fragmentation leading to male and female sterility (Hu et al., 2018). Here, Zhang et al., demonstrate that RAD17 is also essential for meiotic DSB repair in maize but, remarkably and contrary to rice, only in male meiosis. Thus, besides underlining the importance of studying various plant species, this work also points to important differences between male and female meiosis (highlighted by Gutierrez Pinzon et al.). New issues have also recently emerged at the forefront of research on meiotic recombination. First, considering the impact of global warming, understanding how temperature affects meiosis has become a major challenge and recent breakthroughs have been comprehensively described by Gutierrez Pinzon et al. Second, Dziegielewski and Ziolkowski present an extensive review of the knowledge around non-coding RNAs (ncRNAs) and their impact on plant meiosis. NcRNAs are key players in many biological processes, but their role in meiosis has remained elusive. An interesting proposal of Dziegielewski and Ziolkowski is that ncRNA pathways regulate meiosis through the controlled expression of meiosis-specific genes and this role may have evolved as a secondary effect of their primary function in the control of transposable elements in germ cells.

Visualization of meiotic chromosomes has been of major importance for our understanding of meiotic recombination and the dynamics of chromosome behavior. Sims et al. provide an overview of classical and advanced cytological sample preparation methods, and review the latest developments in microscopy techniques from epifluorescence, confocal laser scanning and super-resolution microscopies in Arabidopsis. The authors include representative STED (stimulated emission depletion)-based images of Arabidopsis meiotic proteins immunostained on chromosomes and suggest that nanoscale imaging will help in characterizing the fundamental processes of meiosis. Super-resolution microscopy has already provided us with novel insights into CO interference by determining the location, amount and intensity of the meiotic protein HEI10 E3 ligase in Arabidopsis (Capilla-Pérez et al., 2021; Morgan et al., 2021a). As an alternative approach of visualizing plant chromosomes, Prusicki et al. review technical aspects and applications of live imaging of meiosis in plants. The development of novel genomic approaches has also advanced our understanding of meiosis. For instance, Barr et al. develop an INTACT system to purify meiotic nuclei in a high-throughput manner in Arabidopsis and discover the importance of DNA demethylation in plant meiosis. The meiocyte INTACT system can be combined with single cell RNA sequencing (Nelms and Walbot, 2019) and other genomic approaches such as ATAC-seq, bisulfite-seq and ChIP-seq for mapping of meiotic chromatin features. Post-translational modifications also play crucial roles in the control of meiosis. Orr et al. highlight recent advances on the roles of ubiquitination in plant meiosis and overview various proteomic approaches for identifying substrates of ubiquitin E3 ligases which include BioID/TurboID-based proximity labeling. The proximity labeling and affinity purification–mass spectrometry can be adapted to generate a wide view of protein interactome during meiosis (Mair et al., 2019; Yang X. et al., 2021).

Along with powerful genetic screening of plant meiotic mutants, these advanced approaches have helped to confirm that COs are not evenly distributed along plant chromosomes. For instance, they are enriched in distal regions but also in interstitial regions that are at junctions with heterochromatin in Arabidopsis. In contrast, COs are almost exclusively restricted to distal regions in cereals. A correlation between CO distribution, transposon content and DNA methylation exists in plant species (Lambing et al., 2017). Raz et al. use Virus-Induced Gene Silencing to down-regulate the expression of genes coding for DNA methylases recombination proteins in tetraploid wheat and show that it is possible to influence the pattern of recombination using non-transgenic approaches. This technique has the potential to facilitate plant breeding by creating novel genetic diversity in regions normally deprived in meiotic recombination. However, in order to profoundly impact future breeding strategies, the control of meiotic recombination remains to be fully understood. Kuo et al. provide an overview on the factors known to be involved in CO distribution and hypothesize that the formation of COs near the telomere is a default position caused by the pairing of the telomeres prior to the initiation of recombination. Aguilar and Prieto extend this concept and review our current knowledge on the dynamics of the telomeres and sub-telomeres. The authors suggest that distal chromosome recognition could play an important role in the correct chromosome pairing in polyploid species. Since the telomeric repeats are highly conserved between plants species, the authors propose that the sub-telomeric regions, rather than the telomeres, may help differentiating homologous from homoeologous pairing. These new models of CO distribution and chromosome pairing will likely drive future experimental investigations. Kuo et al. further propose that a change in the composition of the chromosome axis between Arabidopsis and wheat could be a major contributor to the different patterns of recombination observed between the two species. In support to this model, Osman et al. perform a detailed analysis of meiotic recombination in hexaploid wheat and show that the chromosome axis and DSBs initiate first in distal regions before occurring in interstitial and proximal regions. The authors speculate that the sequential events of meiotic progression have an influence on the position of COs along the chromosomes, with the regions recombining first being more likely to form a CO while the regions recombining last rarely recombine. This recombination pattern is also influenced by an interfering signal that initiates at the CO sites and inhibits the formation of additional COs in adjacent regions. The formation of a CO involves the linkage between two chromatids from each of the two homologous chromosomes. It had remained unknown if interference can spread across the chromatids that are not directly involved in the CO. To answer this long-standing question, Sarens et al. develop a novel approach to quantify chromatin interference. The authors found that the interfering signal represses the formation of a second CO on the two chromatids of each chromosome and concluded that CO interference acts on the whole chromosome. In a separate study, Morgan et al. (2021b) showed that CO interference occurs along multiple connected axes to repress the formation of multivalent connections in tetraploid *Arabidopsis arenosa*.

One of the most important challenges in meiosis arises after whole genome duplication (WGD). The presence of more than two chromosome sets in the same meiosis may lead to the formation of univalents and multivalents during prophase I and subsequent chromosome mis-segregation during anaphase I. To face these problems, polyploids have developed strategies to control pairing preferences that result in diploid-like behavior during meiosis and disomic inheritance. Svačina et al. use allohexaploid bread wheat as a model to review molecular mechanisms and regulators involved in maintaining diploid-like pairing behavior in allopolyploids (polyploids resulted from the hybridization of related species). WGD is a prominent evolutionary process relevant for crop improvement. Indeed, many cultivated plants such as wheat, tobacco, potato, cotton, or sugarcane, among others, are polyploids. In addition, polyploids often display better tolerance to abiotic stresses (Van de Peer et al., 2021). Natural polyploids may emerge through several pathways, described in detail by Svačina et al., with the generation of unreduced gametes being the more predominant. The production of these gametes, although highly influenced by the environment, also has a genetic basis (Van de Peer et al., 2017). The presence of mutations in certain genes may have contributed to polyploidisation, facilitating the formation of unreduced gametes by defects in either meiosis I or II. Recently, it has been reported that the function of the STRUCTURAL MAINTENANCE OF CHROMOSOME 5/6 (SMC5/6) complex is essential to ensure accurate gametophytic ploidy in Arabidopsis (Yang F. et al., 2021). Mutants defective for this complex generate unreduced gametes by recombination-independent mechanisms and produce triploid offspring. Yang et al. analyzed autotetraploid plants deficient for SMC5/6 and found even more drastic meiotic defects than in diploids, highlighting the importance of this complex in the maintenance of tetraploid genome stability. The meiotic function of other SMC complexes (cohesin, condensin) and associated cofactors, also involved in genome maintenance, is reviewed by Bolaños-Villegas. Besides polyploidy, holocentricity is another challenge to the proper progression of meiosis in the evolution of several plant species. Holocentric chromosomes possess multiple kinetochores dispersed along their length rather than a single region that functions as the centromere. As well as chromosome duplication, holocentric chromosomes evolved several times during plant evolution (Mandrioli and Manicardi, 2020). In plants, the presence of holocentric chromosomes is linked to inverted meiosis, a meiosis with a reverse order in which segregation of homologous chromosomes occurs during meiosis II. In an interesting review, Hofstatter et al. describe adaptations during meiosis in holocentric plants.

Overall, our Research Topic provides an in-depth overview of the latest developments in meiosis and will be of interest to a broad readership on meiosis, genome evolution and plant breeding.

## Author Contributions

All authors listed have made a substantial, direct, and intellectual contribution to the work and approved it for publication.

## Conflict of Interest

The authors declare that the research was conducted in the absence of any commercial or financial relationships that could be construed as a potential conflict of interest.

## Publisher's Note

All claims expressed in this article are solely those of the authors and do not necessarily represent those of their affiliated organizations, or those of the publisher, the editors and the reviewers. Any product that may be evaluated in this article, or claim that may be made by its manufacturer, is not guaranteed or endorsed by the publisher.
